# 37 Fluorescence Imaging-Guided Sampling Provides More Accurate and Actionable Microbiology Compared to the Levine Technique

**DOI:** 10.1093/jbcr/iraf019.037

**Published:** 2025-04-01

**Authors:** Erik Hanson Viana

**Affiliations:** Hospital Angeles

## Abstract

**Introduction:**

Swab sample collection to rule out infections pre-grafting is challenging in larger wounds where sparse microbial clusters may be missed. Fluorescence (FL) imaging technology enables real-time detection of bacteria/biofilm, and studies have linked FL signals in the wound bed to the failure of autografts and xenografts. We hypothesized that bedside FL imaging would provide more accurate pre-operative wound assessments than standard methods, thereby improving graft timing and placement for better patient outcomes.

**Methods:**

Prospective observational diagnostic study where FL-imaging is compared to the SOC as guidance for sample site selection prior to Split Thickness Skin Graft (STSG) or dermal regeneration template (DRT) application. Microbiology determines the timing of the STSG. All patients had been previously infected, treated and were deemed clinically negative prior to the sampling procedure. Power analysis yielded a minimum sample size of 50 participants. Pre-operative standard of care (SOC) swabbing (Levine technique) was performed by the surgical team, while FL-guided samples were collected independently at the same time by an independent researcher. Statistical analysis included Chi-squared and Mc Nemar’s tests to compare the pathogens identified by the two sampling methods, and to evaluate the methods’ proportion of true positives/negatives.

**Results:**

76 burn patients were enrolled. 76% of the cohort had burns involving >20% TBSA. Most SOC samples came back negative (56/76), and of those that were positive, commensal bacteria was reported as the only or main microbe in 56%. FL-guided samples captured significantly more positive samples (54/76). The sensitivity and specificity of FL-guided samples was 87% and 96% compared to 46% and 85% for SOC, respectively. When considering Pseudomonas specifically, sensitivity was higher for FL-guided samples (93% FL vs 46% SOC). Notably, NPV of FL was 92% for all kinds of bacteria compared to 71% among SOC swab cultures.

**Conclusions:**

Pre-operative burn wound sampling guided by FL imaging identified more relevant pathogens with greater sensitivity compared to the SOC Levine technique. Specifically, Pseudomonas detection sensitivity improved by 48% with FL guidance. FL imaging seems to provide higher predictive value for determining the appropriate time for grafting, offering timely results over negative swab cultures.

**Applicability of Research to Practice:**

Objective, FL-targeted sampling of large burn wounds may better inform graft timing decisions for optimized grafting outcomes.

**Funding for the Study:**

None to declare. The FL-imaging device was loaned at no cost by the local distributor.

